# Development of chromatographic methods to determine multivitamins formulation depending on their solubility and polarity: comparative study using three greenness assessment tools

**DOI:** 10.1186/s13065-024-01118-1

**Published:** 2024-01-27

**Authors:** Sara Abdel Basset Galal, Eman Saad Elzanfaly, Emad Mohamed Hussien, Enas Abdel Hakim Amer, Hala Elsayed Zaazaa

**Affiliations:** 1 Egyptian Drug Authority, 51 Wezaret El- Zeraa Street, Agouza, Giza, 12618 Egypt; 2 Analytical Chemistry Department, Faculty of Pharmacy, Cairo University, Kasr El- Aini Street, Cairo, 11562 Egypt

**Keywords:** Ipriflavone, Ascorbic acid, Pyridoxine HCl, Vitamin D3, Lysine HCl, RP-HPLC method, Greenness Assessment

## Abstract

High performance liquid chromatography is one of the techniques of choice for the separation and quantitative determination of drugs in mixture form. Ipriflavone, ascorbic acid, pyridoxine, vitamin D3, and lysine are formulated together as an adjuvant combination in osteoporosis**.** In this work, we developed and validated two complementary high performance liquid chromatographic methods to determine the five compounds in their pharmaceutical dosage form. The first method (method A) was capable of determining ipriflavone, ascorbic acid, pyridoxine, and vitamin D3 in their bulk and combined pharmaceutical formulation. The method is based on Liquid Chromatographic separation with UV detection at 254 nm using Agilent Eclipse XDB-C18 column with a mobile phase consisting of 25 mM ammonium acetate buffer (pH 4.2): methanol in gradient mode. Due to the high polarity of lysine, it was difficult to achieve satisfactory retention on reversed phase columns. So, we separated it on a strong cation exchange column (Exsil 100 SCX) without derivatization with a mobile phase consisting of 10 mM sodium dihydrogen phosphate and 200 mM sodium chloride (pH 6) with UV detection at 210 nm (method B). Validation of the proposed methods was performed according to ICH guidelines Q2(R1). The proposed methods proved to be valid for selective analysis of the stated drugs in their bulk and combined pharmaceutical formulation. Greenness assessment of the developed methods was evaluated using three assessment tools: ESA, GAPI and the most recently developed tool AGREE, showing a satisfactory comprehensive guide of the greenness of the developed methods.

## Introduction

**Fig. 1:**
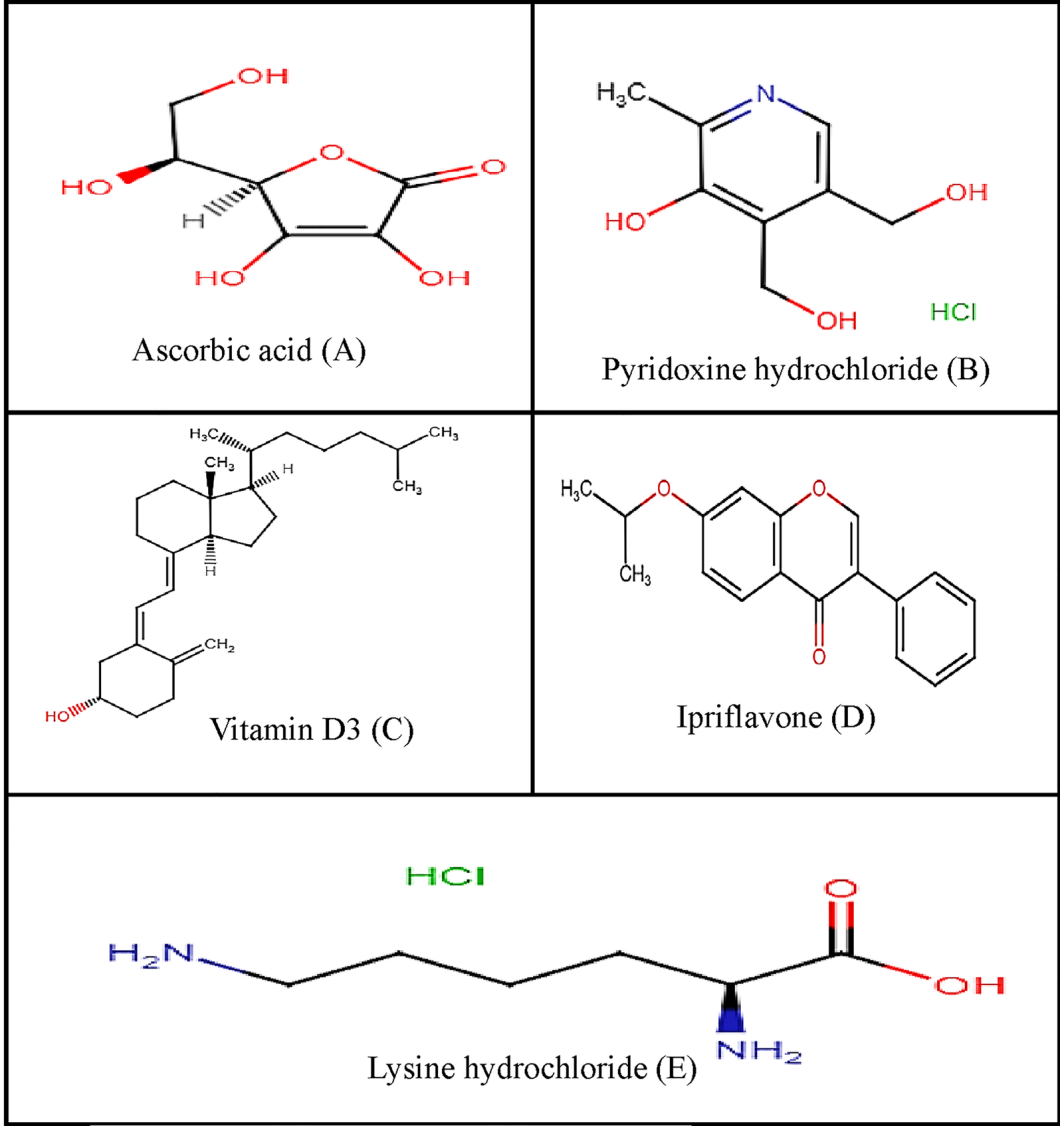
Chemical structures of active drug substances

Ascorbic acid (vitamin C) (Asc) (Fig. 1A) plays a chief role as a redox cofactor and catalyst in a broad range of biochemical reactions and processes [1]. Pyridoxine hydrochloride (Py) (Fig. 1B) is 4,5-bis(hydroxymethyl)-2-methylpyridin-3-ol hydrochloride. Among the B vitamins, pyridoxine hydrochloride (vitamin B6) has unique role in the metabolism of lipids, proteins, and carbohydrates. Vitamin D3(Vit D3), (Fig. 1C) is (5Z,7E) -(3S)-9,10-seco-5,7,10(19)-cholestatrien-3-ol, it acts to maintain calcium and phosphorus homeostasis which is required for many biological processes. Ipriflavone (Ip) (Fig. 1D) is 7-isopropoxy-3-phenyl-4H-chromen-4-one [2], it is used for the prevention and treatment of postmenopausal osteoporosis. Ipriflavone is effective in the reduction of bone turnover by inhibiting bone resorption and stimulating bone formation [3]. Lysine hydrochloride (Lys) (Fig. 1E) is an essential amino acid used as building unit for proteins in the body.

The studied active drug substances are formulated together in a capsule form that is used as an adjuvant therapy in case of loss of bone density.

Several methods including HPLC with UV, FLD and/or mass detection [4–20], micellar electrokinetic chromatographic (MEKC) [21], spectrofluorimetric [22] were reported for the determination of these compounds in different multivitamins combinations in different matrices.

Ipriflavone was also determined by several techniques, in tablets by square-wave adsorptive cathodic stripping (SW-AdCS) voltammetry and LC- UV methods [23], in plasma by Liquid chromatography with mass detection [24, 25], with its synthetic impurities by HPLC–PDA [26], HPLC -UV study of its pharmacokinetics and distribution in rabbits and human beings [27–30] and also in rat plasma, urine and tissue homogenate [31].

Lysine was analysed by several HPLC methods either singly or in presence of other amino acids using an aqueous mobile phase pumped at low flow rate to achieve satisfactory retention on reversed phase column [32] or by derivatization with ortho-phtaldehyde and 9-Fluorenylmethyl chloroformate [33], 4-dinitro-fluorobenzene (FDNB) [34, 35], or by HILIC column [36].

The concept of green analytical chemistry (GAC) has been emerging recently as a part of the global consideration and concern towards minimizing the hazardous impacts on the environment and health. It aims mainly at developing eco-friendly, less occupationally hazardous analytical methods based on the 12 principles of GAC proposed by Anastas and Warner [37, 38]. Since the evolvement of the GAC principles, many assessment tools and metrics were proposed to evaluate the greenness of the developed analytical methods. These greenness evaluation metric systems vary in the degree of embodiment of more or less of the GAC principles.

Our literature survey revealed that no method was reported to resolve the mixture of the five components or to determine them in their combined dosage form. So, the aim of this work was to develop and validate a HPLC method that can separate Ipriflavone, ascorbic acid, pyridoxine HCl, vitamin D3 and lysine HCl in a reasonable run time to be used for the quality control of their bulk powders and pharmaceutical dosage form. We also aimed to investigate the compliance of the developed methods with the GAC principles using three greenness assessment tools to give a comprehensive overview of the greenness of the proposed methods.

## Experimental

### Materials

**a) Pure samples**

Ipriflavone (99.64%), ascorbic acid (100.17%), pyridoxine hydrochloride (99.8%), vitamin D3 oil 40,000,000 IU/gm and lysine hydrochloride (99.66%) were kindly obtained from Eldebeiky Pharma (DBK), Egypt.

**b) Market samples**

Ultracalce^®^ capsules labeled to contain ipriflavone 25 mg, ascorbic acid 30 mg, pyridoxine hydrochloride 1 mg, vitamin D3 0.005 mg and lysine hydrochloride 25 mg per capsule manufactured by AL-Debeiky Pharma Co. were purchased from the local market.

**c) Chemicals and reagents**

HPLC grade methanol (Scharlau, Spain), ammonium acetate (Qualikems, India), glacial acetic acid (Merck, Germany), sodium dihydrogen phosphate, sodium chloride and phosphoric acid (Merck, Germany) and Merck Milli Q Ultrapure Water were used in chromatographic analyses. Twenty-five millimole ammonium acetate buffer was prepared by dissolving 1.93 g of ammonium acetate in one litre of water (pH was adjusted to 4.2 with glacial acetic acid). The mobile phase for lysine hydrochloride elution was prepared by dissolving 1.56 gm sodium dihydrogen phosphate and 11.9 gm sodium chloride in one litre water (pH was adjusted to 6 with orthophosphoric acid).

### Instrumentation

Dionex ultimate 3000 Standard LC System equipped with a quaternary pump, standard manual injector with a 20 µL loop, multiple wavelength detector with Data acquisition performed on chromeleon 7.2 software was used as chromatographic system.

Sartorius electric balance (Germany). Hamilton microsyringe 100 µL capacity, Sonicator (R. Espinar S.L,Spain) and Digital PH meter (HANNA, USA) were also used.

### Chromatographic conditions

#### Method A

For Ipriflavone, ascorbic acid, pyridoxine hydrochloride, vitamin D3, separation was performed on a reversed phase column, Agilent Zorbax Eclipse XDB-C18 (250 X 4.6 mm i.d, 5 µm particle size), with a mobile phase consisting of solution A (acetate buffer) and solution B methanol for elution of ipriflavone, ascorbic acid, pyridoxine hydrochloride and vitamin D3 in gradient mode with as follows, initially 97% of Solution A (0–3 min), (97–0%) solution A (3–5 min), 0% solution A (5–9 min) then (0–97%) solution A (9–11 min) with three minutes equilibration at the initial conditions between runs. UV detection was performed at 254 nm. The flow rate was 1 mL/min.

#### Method B

For lysine hydrochloride, separation was performed on Dr.Maisch Exsil 100 SCX (150 X 4.6 mm i.d, 5 µm particle size) column using a mobile phase consisting of 10 mM sodium dihydrogen phosphate and 200 mM sodium chloride (pH 6) pumped at flow rate 1 mL/min and detection was performed at 210 nm.

In both chromatographic methods the system was operated at ambient temperature. All samples were filtered through a 0.45 µm syringe filter, and  L were injected by the aid of Hamilton microsyringe. The columns used were conditioned for at least 30 min or until stable baseline before injection.

### Preparation of standard stock and working solutions

Standard stock solutions were prepared in water for Asc, Py and Lys and in methanol for Ip and Vit D3 by accurately weighing 50 mg of each into five 50 mL volumetric flasks (final concentration 1000 µg/mL for all). Further dilutions of standard stock solutions were done into five 100 mL volumetric flasks with the same solvents to prepare working standard solutions of 100 µg/mL for Asc, Py, Ip, Vit D3, and 200 µg/mL for Lys.

### Procedures

#### Construction of calibration curve

Dilutions for calibration curves' construction were prepared by transferring aliquots of Asc, Py, Lys, Ip and Vit D3 working standard solutions equivalent to 25–800 µg, 75–900 µg, 400–2000 µg, 100–1000 µg, 10–600 µg, respectively into series of 10 mL volumetric flasks. The volume was completed to the mark with water in case of Asc, Py and Lys and with methanol in case of Ip and Vit D3 to reach final concentration ranges of 2.5–80 µg/mL for Asc, 7.5–90 µg/mL for Py, 40–200 µg /mL for Lys, 10–100 µg/mL for Ip, and 1–60 µg/mL for Vit D3. Each solution was injected in triplicates and chromatographed under the previously mentioned chromatographic conditions. The chromatograms were recorded, the peak areas were determined and the calibration curve relating peak areas to the corresponding concentrations for each compound was constructed.

#### Application to pharmaceutical formulations

The content of ten capsules were mixed well, the average content weight of one single capsule was weighed and transferred into two 25 mL volumetric flasks. The content of the first flask was dissolved in 15 mL water while the content of the second flask was dissolved in 15 mL methanol by sonication in ultrasonic water bath for 10 min. The volume was completed to the mark with the same solvents in each flask. From the stock solution in water, 1.25 mL was diluted into 75 mL volumetric flask (Asc preparation), 1.25 mL into 5 mL volumetric flask (Py preparation), and 0.4 mL into 10 mL volumetric flask (Lys preparation). In all the three preparations the volume was completed to the mark with water to get final nominal concentrations 20 µg/mL, 10 µg/mL, 40 µg/mL for Asc, Py, Lys, respectively.

From the stock solution in methanol, for Ip sample solution 0.5 mL was diluted into 25 mL volumetric flask and the volume was completed to the mark with methanol (final nominal concentration for Ip 20 µg/mL). Vit D3 was determined by weighing one capsule fill weight into 5 mL volumetric flask, dissolving in methanol with sonication in ultrasonic water bath for 5 min (final nominal concentration 1 µg/mL). Standard addition technique [40] was done to confirm results due to the very low concentration of Vit D3 in capsule (5 µg/capsule).

## Results and discussion

### Method optimization

#### Optimization of mobile phase

**Fig. 2:**
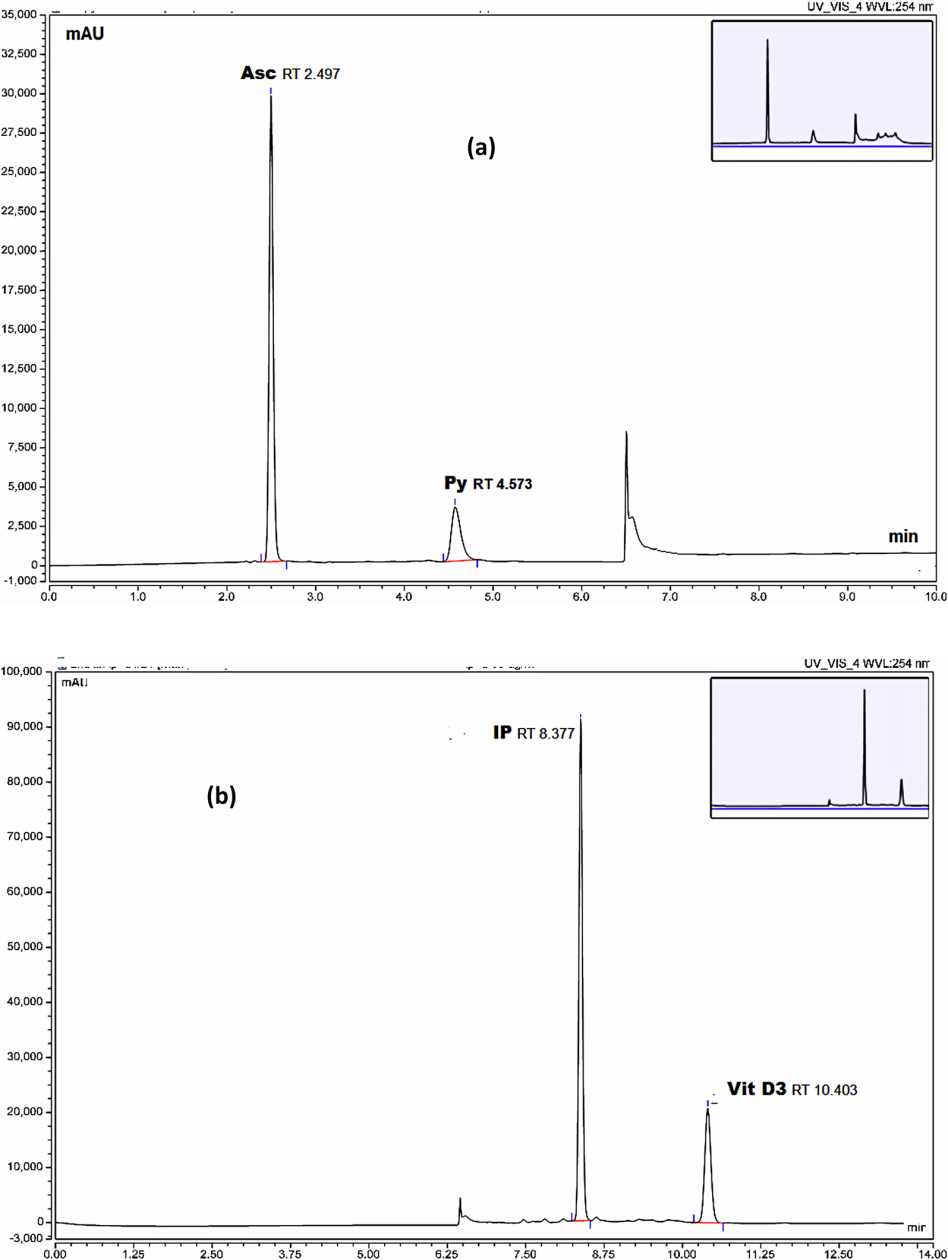
**a** HPLC chromatogram for separation of Asc and Py. **b** HPLC chromatogram for separation of IP and Vit D3

We made many attempts to separate the five analytes together in one single run using many different mobile phases' compositions like water and acetonitrile in different proportions, buffers including TEAC (triethyl amine: acetic acid mixture), phosphate buffer with changing pH values and compositions with different organic modifiers but in most cases lysine was not observed and no complete satisfactory separation and resolution between the other four compounds (Asc, Py, IP, and Vit D3) were achieved. Finally, acetate buffer with methanol combination was tried in gradient mode due to the good solubility of ammonium acetate in methanol where we achieved good separation and resolution between the four compounds in reasonable run time but still lysine was not detected. Two injections were done one for the two analytes dissolved in water (Asc, Py) and second run for the two analytes dissolved in methanol (IP and Vit D3) (Fig. 2).

**Fig. 3:**
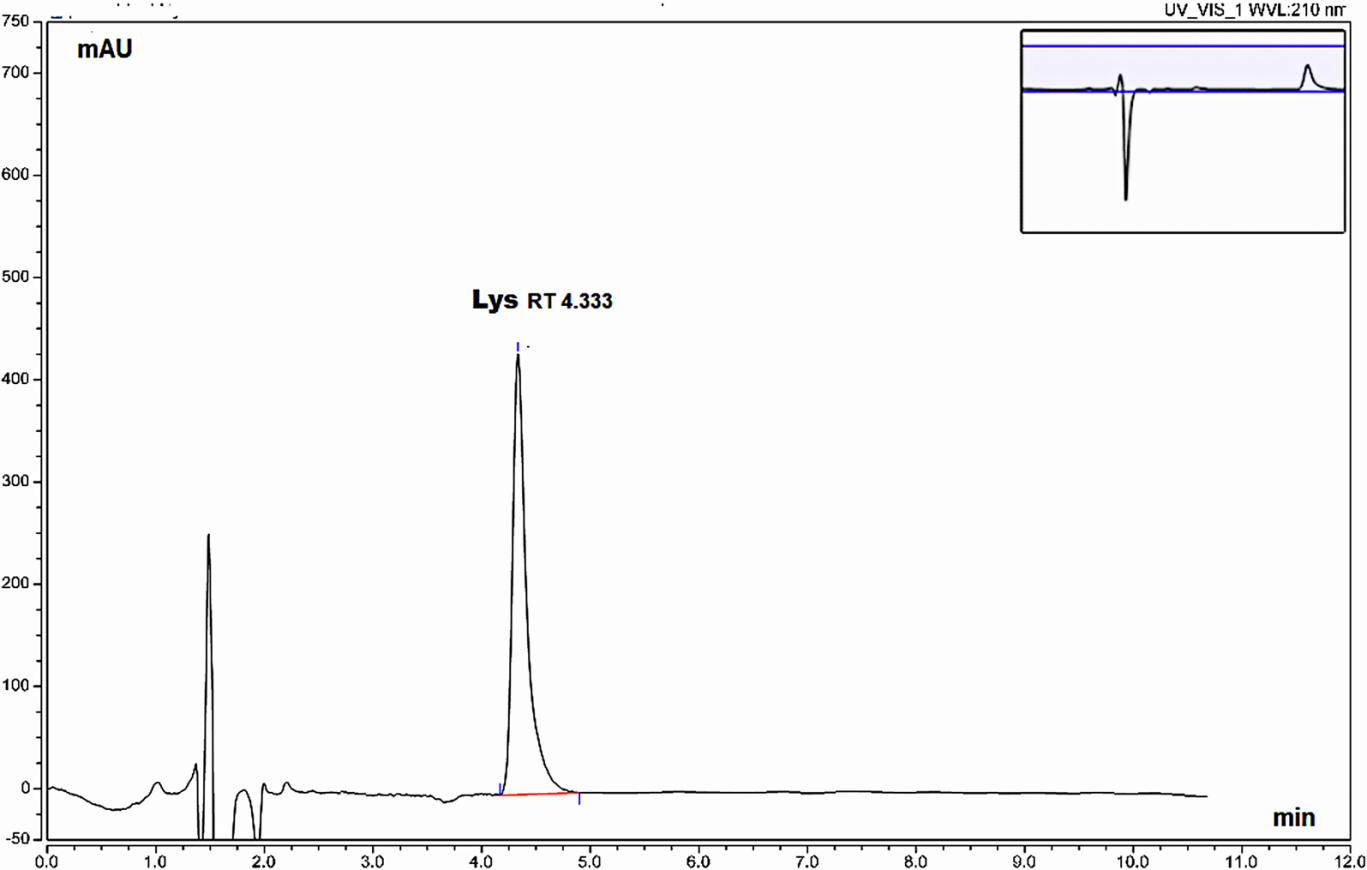
HPLC chromatogram for separation of Lys using the cation exchange column

**Fig. 4:**
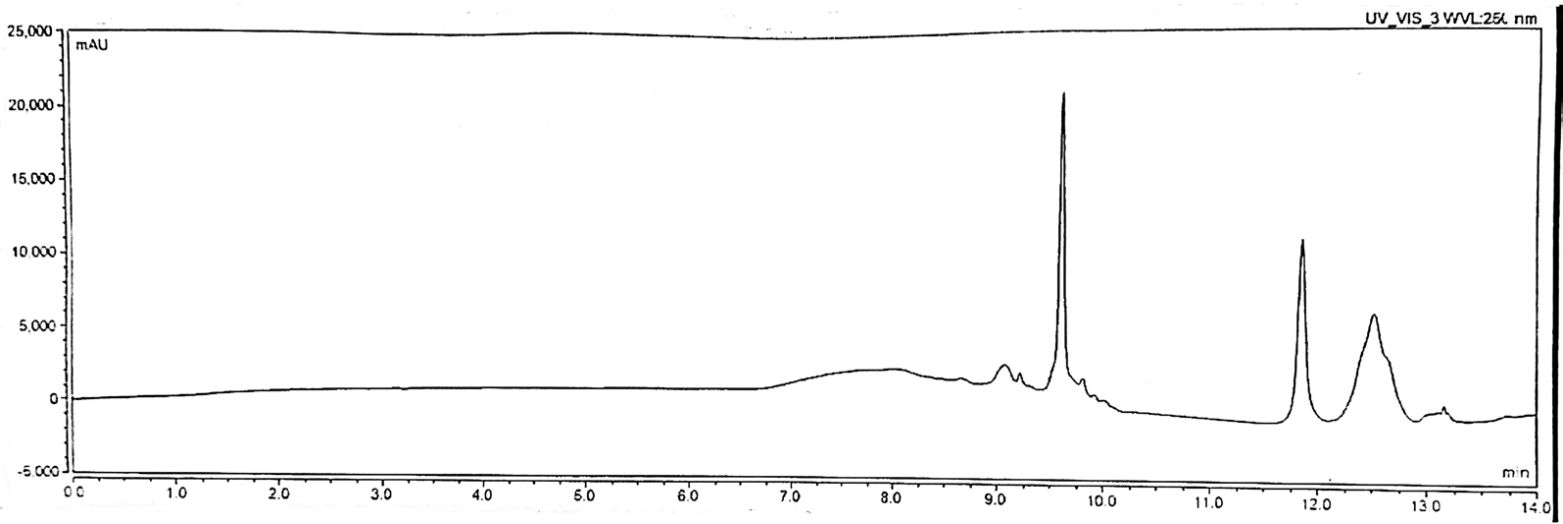
Blank injection chromatogram for method A

**Fig. 5:**
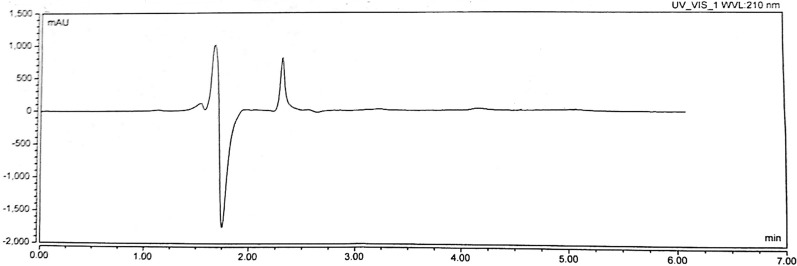
Blank injection chromatogram for method B

In all trials on different reversed phase columns (C8, C18), it was difficult to achieve satisfactory retention for lysine due to its high polarity. In an attempt to comply with GAC principles, we avoided its determination by the tedious derivatization approach to reduce the use of reagents, so we considered another mode of HPLC separation depending on lysine polarity and its basic nature on a strong cation exchange column. Strong cation exchange columns with aliphatic sulfonic acid groups carry negative charges in all pH ranges, therefore can bind basic analytes and this mode is widely used in the separation of a range of molecules from amino acids to large biomolecules where elution is achieved by increasing ionic strength of the mobile phase with high salt concentration. Under these conditions, we obtained good retention and separation for Lys (Fig. 3). Figures 4 and 5 show blank injections.

#### Optimization of gradient mode

Due to the polar nature of Asc and Py, they were separated under high aqueous conditions especially for Asc to achieve satisfactory retention on reversed phase column then the organic modifier was linearly increased to elute the highly hydrophobic Vit D3 and IP with good resolution.

#### Selection of buffer pH

For method A, buffer pH is critical for Asc and Py peak shape and elution. Both have two pka values; Asc is a diprotic acid while Py is a basic compound so optimizing the pH to 4.2 after trials of many buffers with many different pH values provided slightly acidic medium for Asc and Py to achieve good retention and resolution. Upon increasing pH value, elution of three peaks was observed probably representing two ionization states of Py. Decreasing pH < 4 caused peak distortion of Py (appearance of peak shoulder). Buffer pH has no effect on the elution of IP and Vit D3 as they have to be eluted under high organic conditions due to their hydrophobic nature and retention on reversed phase columns.

For method B, pH has no effect on the SCX stationary phase as it carries negative charge over the whole pH range, but the pH is critical for the analyte to be bound to the stationary phase. pH 6 was found to be optimum for the elution of Lys. At this pH value Lys is positively charged achieving good retention to the negatively charged stationary phase. Then elution with replacement by salts from the stationary phase gave good peak shape and satisfactory retention relative to the standard reversed phase mode of separation.

#### Selection of detection wavelength

For method A, the maximum wavelengths were 265 nm for Asc, 220 nm for Py, 250 nm for IP, 212 and 265 nm for Vit D3, so we chose 254 nm as a center point to allow the determination of the four analytes at single wavelength with reasonable sensitivity for the four compounds.

For method B, Lys lacks chromophores, so it should be determined at short wavelengths to achieve good sensitivity so 210 nm was found to be optimum for its determination with good sensitivity.

#### Selection of type of solvents

We chose water to dissolve Asc, Py and Lys as they are all freely soluble in it. Also, methanol could not be used under almost 100% aqueous mobile phase at the start of the run to avoid peak shape deforming due to solvent elution strength exceeding that of the mobile phase. Methanol was chosen to dissolve IP and Vit D3 as both are freely soluble in it. Selection of solvent is especially crucial for Vit D3 as it is present in very low dose in capsule (5 µg/cap) so solvent in which it is freely soluble is mandatory to extract Vit D3 completely from capsule for accurate determination.

### Method validation

The proposed method was validated according to the ICH guidelines [39, 40].

#### System suitability

The system suitability parameters are presented in (Table 1) including capacity factor which is a measure of the location of the peak of interest with respect to the void volume and column efficiency expressed as number of theoretical plates (N) which represents the number of peaks that can be eluted per unit run-time of the chromatogram [40]. All parameters were satisfactory and met the recommended acceptance criteria.

**Table 1:** System suitability testing parameters for the developed HPLC methods

Parameters	Method A				Method B	
	Ascorbic acid	Pyridoxine hydrochloride	Ipriflavone	Vitamin D3	Lysine hydrochloride	Reference value (40)
Tailing factor (T)	1.12	1.46	0.99	1.05	1.55	≤ 2
Capacity factor (K)	2.13	4.7	9.47	12.12	3.23	> 2
Column efficiency (N)	13660	7810	81944	47510	6656	> 2000
Resolution (Rs)**	14.19		13.73		–	> 2

^**^Calculated between each compound and the nearest peak

#### Linearity

The linearity of the developed method was investigated, and the linear regression data for the calibration curves showed good linearity with correlation coefficient (r) > 0.999.

#### Range

The specified ranges derived from linearity studies were over the following concentrations: 2.5–80 µg/mL, 7.5–90 µg/mL, 10–100 µg/mL, 1–60 µg/mL, 20–200 µg/mL for Asc, Py, Ip, Vit D3, Lys, respectively with respect to peak area (Table 2).

**Table 2:** Validation parameters of the proposed HPLC methods for the determination of Ipriflavone, Ascorbic acid, Pyridoxine, Vitamin D3 and Lysine

Parameters	Value				
	Ascorbic acid	Pyridoxine hydrochloride	Ipriflavone	Vitamin D3	Lysine hydrochloride
Wavelength (nm)	254 nm	254 nm	254 nm	254 nm	210 nm
Linearity range	2.5–80 µg/mL	7.5–90 µg/mL	10–100 µg/mL	1–60 µg/mL	20–200 µg/mL
Time of analysis (min/run)	14 min				6 min
Regression equation (Y = bX ‏ + a)*	Y = 51.229 X-6.3213	Y = 7.8682X + 14.192	Y = 118.9 X + 84.916	Y = 45.711 X + 1.5154	Y = 0.5756 X + 0.2469
Intercept (a)	6.3213	14.192	84.916	1.5154	0.2469
Slope (b)	51.229	7.8682	118.9	45.711	0.5756
Correlation coefficient (r)	0.9999	0.9997	1	0.9998	0.9998
Accuracy (mean ± %RSD)	100.53% ± 1.33	100.27% ± 1.44	99.76% ± 1.28	100.8% $\pm$ 0.72	99.95% ± 1.41
Precision (% RSD)					
Repeatability^a^	1.33	1.44	1.28	0.72	1.41
Intermediate precision^b^	1.22	1.27	1.3	1.07	1.16
Average recovery of the content of drug substances in Ultrcacalce^®^capsules					
(Mean ± RSD)	32.86% ± 1.58^c^	93.45% ± 1.16	95.31% ± 0.87	107.67% ± 1.57	106.88% ± 1.66

^*^Y is the analytical signal and X is the concentration

^a^The intra-day (n = 9) average of three different concentrations repeated 3 times within 1 day

^b^The inter-day (n = 9) average of three different concentrations repeated 3 times in 3 successive day

^c^May be due to improper storage conditions

#### Accuracy

Accuracy of the proposed methods was studied by standard addition technique on true solution and on pharmaceutical formulation and evaluating recovery results. Results presented in Tables 2, 6, 7, 8, 9, 10 indicate good accuracy of the proposed methods (Recovery at each level ± RSD: 100.00% ± 2).

#### Precision

We analysed homogenous samples of capsules over different days to obtain inter-days (intermediate precision, n = 3 for each concentration) and within the same day to obtain intra-day precision (repeatability, n = 3 for each concentration), then the RSDs % values were calculated. The developed method was proved to be precise as the RSD% was < 2% (Table 2).

#### Robustness

Robustness of the method was checked by investigating the effect of small deliberate changes in the experimental conditions on the system suitability parameters. We evaluated the change in system suitability parameters upon elution of analytes with mobile phase of minor change in pH value (4.2 ± 0.2) and different column brands. The RT values of the four analytes and resolution between them using the mentioned pH range did not change, while changing the column brand was accompanied by slight decrease or increase of RT of the eluted peaks also minor increase and decrease in resolution between them. For method B for separation of Lys, robustness was evaluated upon change in mobile phase pH and flow rate with satisfactory corresponding change in system suitability parameters (Tables 3, 4, 5).

**Table 3:** Robustness study for the developed HPLC method for the determination of Ipriflavone, Ascorbic acid, Pyridoxine and Vitamin D3 upon change in pH

Parameter	Change in pH											
	pH 4				pH 4.2				pH 4.4			
System suitability parameter	K	Tailing factor	Theoretical plates	Rs*	K	Tailing factor	Theoretical plates	Rs*	K	Tailing factor	Theoretical plates	Rs*
Ascorbic acid	2.2	1.1	12698	14.22	2.11	1.09	13660	14.19	2.15	1.14	14505	14.15
Pyridoxine hydrochloride	4.63	1.5	7946	–	4.73	1.47	7810	–	4.7	1.49	8210	–
Ipriflavone	9.5	1.02	85490	13.68	9.52	0.99	81944	13.73	9.48	0.99	77579	13.78
Vitamin D3	11.9	1.1	47407	–	12.11	1.05	47510	–	12.2	1.06	48110	–

^*^Calculated between each compound and the nearest peak

**Table 4:** Robustness study for the developed HPLC method for the determination of Ipriflavone, Ascorbic acid, Pyridoxine and Vitamin D3 upon change in column brand

Parameter	Change in column brand							
Column brand	Agilent Eclipse XDB-C18				Thermo hypersil BDS C18			
System suitability parameter	K	Tailing factor	Theoretical plates	Resolution*	K	Tailing factor	Theoretical plates	Resolution*
Ascorbic acid	2.1	1.09	13660	14.19	2.05	1.19	9651	15.8
Pyridoxine hydrochloride	4.7	1.47	7810	–	5.07	1.02	8790	–
Ipriflavone	9.5	0.99	81944	13.73	8.39	1.84	86875	12.57
Vitamin D3	12.1	1.05	47510	–	10.5	1.64	49834	–

^*^Calculated between each compound and the nearest peak

**Table 5:** Robustness study for the developed HPLC method for the determination Lysine upon change in pH value and flow rate of the mobile phase

Parameters	Change in flow rate								
	Flow 0.9 mL/min			Flow 1 mL/min			Flow 1.1 mL/min		
Lysine hydrochloride	K	Tailing factor	Theoretical plates	K	Tailing factor	Theoretical plates	K	Tailing factor	Theoretical plates
	3.1	1.67	6834	2.85	1.55	6656	2.6	1.55	6373
	Change in pH								
	pH 5.8			pH 6			pH 6.2		
	K	Tailing factor	Theoretical plates	K	Tailing factor	Theoretical plates	K	Tailing factor	Theoretical plates
	2.71	1.42	8106	2.85	1.55	6656	2.85	1.52	6493

#### Specificity

The specificity of the proposed methods was examined for the presence of interference from excipients or sample matrix by applying the standard addition on the dosage form to which known amounts of each analyte have been added to give good recovery results confirming no interference from other excipients or analytes in capsule matrix [40] (Tables 6, 7, 8, 9, 10).

**Table 6:** Evaluation of the accuracy of the proposed HPLC method (method A) for the determination of Ascorbic acid by standard addition on Ultracalce^®^capsules

Claimed Conc of Asc taken from capsule in (µg/mL)	%Found Conc. ± RSD*	Std. Conc. Added (µg/mL)	Total recovered Conc(µg/mL)	Recovered Conc of standard added (µg/mL)	% Recovery
20	32.86% ± 1.58	10.00	16.73	10.16	101.6
		20.00	26.32	19.75	98.75
		40.00	46.50	39.93	99.83

*Average of three determinations

**Table 7:** Evaluation of the accuracy of the proposed HPLC method (method A) for the determination of Pyridoxine hydrochloride by standard addition on Ultracalce®capsules

Claimed Conc of Py taken from capsule in (µg/mL)	%Found Conc. ± RSD*	Std. Conc. Added (µg/mL)	Total recovered Conc(µg/mL)	Recovered Conc of standard added (µg/mL)	% Recovery
10	93.45% ± 1.16	10.00	19.23	9.88	98.8
		20.00	29.37	20.02	100.1
		30.00	39.44	30.09	100.3

*Average of three determinations

**Table 8:** Evaluation of the accuracy of the proposed HPLC method (method A) for the determination of Ipriflavone by standard addition on Ultracalce^®^capsules

Claimed Conc of Ip taken from capsule in (µg/mL)	%Found Conc. ± RSD*	Std. Conc. Added (µg/mL)	Total recovered Conc(µg/mL)	Recovered Conc of standard added (µg/mL)	% Recovery
20	95.31% ± 0.87	10.00	29.1	10.04	100.4
		20.00	39.23	20.17	100.85
		40.00	59.81	40.75	101.88

*Average of three determinations

**Table 9:** Evaluation of the accuracy of the proposed HPLC method (method A) for the determination of Vitamin D3 by standard addition on Ultracalce^®^capsules

Claimed Conc of Vit D3 taken from capsule in (µg/mL)	%Found Conc. ± RSD*	Std. Conc. Added (µg/mL)	Total recovered Conc(µg/mL)	Recovered Conc of standard added (µg/mL)	% Recovery
1	107.67% ± 1.57	2	3.09	2.01	100.5
		5	6.11	5.03	100.6
		15	16.28	15.2	101.33

*Average of three determinations

**Table 10:** Evaluation of the accuracy of the proposed HPLC method (method B) for the determination of Lysine hydrochloride by standard addition on Ultracalce®capsules

Claimed Conc of Lys taken from capsule in (µg/mL)	%Found Conc. ± RSD*	Std. Conc. Added (µg/mL)	Total recovered Conc(µg/mL)	Recovered Conc of standard added (µg/mL)	% Recovery
40	106.88% ± 1.66	20	63.15	20.4	102
		40	82.9	40.15	100.38
		80	124.26	81.51	101.89

#### Analysis of marketed formulation (Ultrcacalce®capsules)

**Fig. 6:**
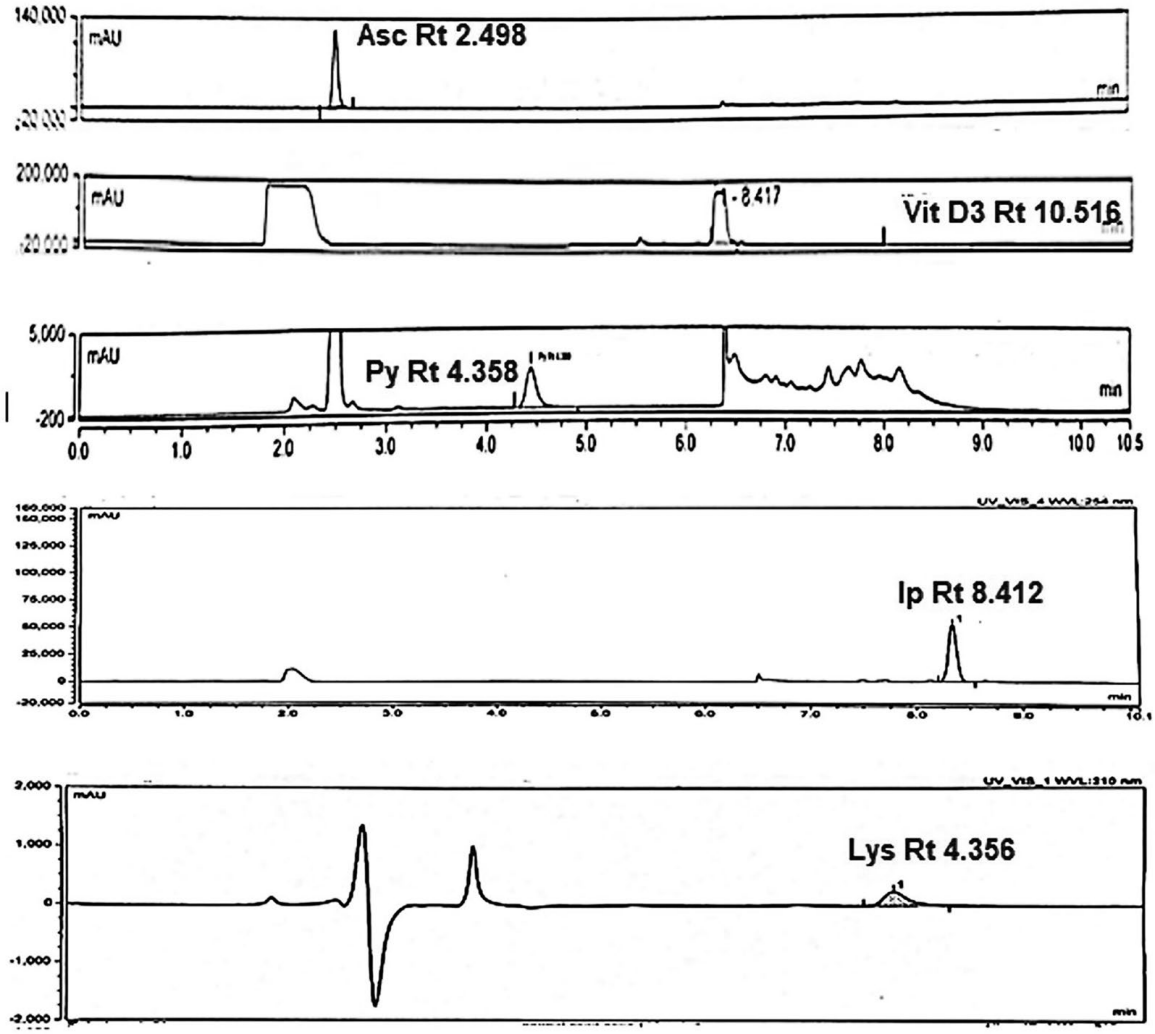
chromatograms of different preparations of Ultracalce® capsule

The proposed HPLC methods were successfully applied for the quantitative assay of the five compounds in its pharmaceutical formulation with good percentage recoveries (Tables 2, 6, 7, 8, 9, 10). Chromatograms for pharmaceutical formulation preparations are shown in Fig. 6

### Greenness assessment

In our attempt to stick to the global commitment to imply greenness in any developed procedures, we assessed the greenness of the proposed methods before proceeding for method validation and application. Three metrics were used for greenness assessment namely Analytical Eco-Scale Assessment (ESA) [41, 42], Green Analytical Procedure Index (GAPI) [43], and the last and the newest one: AGREE—Analytical GREEnness metric [44]. Investigation of the methods' greenness by Eco-scale metric shows that both methods have excellent greenness profiles.

GAPI also can give more thorough coverage of the whole analytical procedure starting from sample collection to waste treatment. From the pictograms of the developed methods A and B, it can be observed that the red segments are due to the macroextration scale of pharmaceutical products and the use of methanol representing non-green solvent in the case of method A and the associated health and safety hazards also the slightly large waste generated from HPLC methods. The same data can be reflected in another way in the twelve sections of AGREE pictogram with additional details about sample amount, process automation, and analysis throughput and operator safety. Red parts of the pictograms in the developed methods are due to the manual processing, methanol as a non-green solvent in method A but generally, the two methods can be considered eco-accepted. Results in Tables 11, 12 prove that the proposed methods are eco-friendly.

**Table 11:** Penalty points for greenness assessment of the proposed methods by Eco scale tool

Hazard	Penalty points (Method A)	Penalty points (Method B)
Ammonium acetate buffer	0	–
Sodium dihydrogen phosphate buffer	–	0
Sodium chloride	–	0
Glacial acetic acid	4	–
Ortho-Phosphoric acid	–	4
Methanol	12	
Instruments energy	1	1
Occupational hazard	0	0
Waste	6	6
Total penalty points	23	11
Analytical eco-scale total score	77	89

^*^ > 75 represents excellent green analysis, > 50 represents acceptable green analysis, < 50 represents inadequate green analysis

**Table 12:** AGREE/GAPI assessment of the green profile of the evaluated procedures for determination of Ipriflavone, Ascorbic acid, Pyridoxine,Vitamin D3 and Lysine

Representative pictogram	Method A	Method B
GAPI^a^		
AGREE^b^		

^a^The color of the pictogram parts are green, yellow, and red indicating the low, medium and high environmental impact involved for each step of the procedures

^b^The resultant pictogram has overall score of fraction of unity in the middle of the pictogram with values close to 1 indicating greener procedures and divided into twelve sections with a number in each section corresponding to the criterion under evaluation, the width of each section indicates their importance and the color range from deep green to deep red reflecting the performance of the each criterion

## Conclusion

The proposed HPLC methods provide simple, accurate and reproducible quantitative analysis for the determination of Asc, Py, Ip, Vit D3 and Lys in their pure form and pharmaceutical formulation, without any interference from the excipients so they can be used in routine quality control analysis. Greenness assessment using different assessment tools gives a satisfactory indication of the environmental impact of the developed methods.

## Data Availability

The datasets used and/or analysed during the current study are available from the corresponding author on reasonable request.
